# Retraction Note: Three SAUR proteins SAUR76, SAUR77 and SAUR78 promote plant growth in *Arabidopsis*

**DOI:** 10.1038/s41598-022-06178-8

**Published:** 2022-02-01

**Authors:** Zhi-Gang Li, Hao-Wei Chen, Qing-Tian Li, Jian-Jun Tao, Xiao-Hua Bian, Biao Ma, Wan-Ke Zhang, Shou-Yi Chen, Jin-Song Zhang

**Affiliations:** State Key Lab of Plant Genomics, Institute of Genetics and Developmental Biology, Chinese Academy of Sciences, Beijing, 100101 China

Retraction of: *Scientific Reports* 10.1038/srep12477, published online 24 July 2015

The Editors have retracted this Article. After publication, concerns were raised regarding a number of figure panels, specifically:

- Fig. 4a: partial overlap between the panels for 35S-GFP and 35S-SAUR76-GFP.
- Fig. 9a: duplication of Coomassie blue bands for the SAUR78-FLAG time-course and seedling treatment.

The authors have stated they are unable to locate the original data. The Editors therefore no longer have confidence in the reliability of the data reported in the Article.

None of the authors agree to this retraction.

